# Editorial

**Published:** 1840-03-21

**Authors:** 


### THE MEDICAL EXAMINER.

PHILADELPHIA, MARCH 21, 1840.

WE publish to-day the resolutions which were adopted by several medical societies relative to the death of their respected colleague, Dr. PARRISH. He was a practitioner of medicine at Philadelphia, for a period of thirty-five years, and was for a large portion of the time in full and honourable practice. No member of the profession was more generally esteemed by his patients, as well as by his medical brethren. His tact in recognising, and his skill in treating disease, acquired for him the respect of all who claimed his professional assistance, while the kindness and friendliness of his manner and feeling won their affection.

The remarkable tact possessed by Dr. Parrish in recognising disease, and in devising the best means of treating it, was shown in his own history. In his lectures he was in the habit of referring to his own case, and to some others of a similar kind which had fallen under his notice, as a proof that a tendency to phthisis might be counteracted by abundant exercise in the open air. This practice he steadily pursued; and although it is not the only means of arresting the commencement of phthisis, it is the essential foundation of all treatment. In the management of the epidemic of typhus, which occurred here some years since, and in numerous other cases in which the views of Dr. Parrish were derived solely from vigorous powers of observation, he gave similar proofs of discernment and skill.

He attained this knowledge of the nature of these diseases, chiefly from his native powers of observation. He was well instructed in his professional studies, but, like many physicians who are pressed upon by the cares of an extended practice, he had not leisure to keep himself familiarly acquainted with the later researches in the pathology of disease. Of the importance of this study he was, however, perfectly aware, and from a conscientious conviction of the duty of a physician to encourage the prosecution of pathological researches, he gave repeated directions that his body should be examined in such a way as would most conduce to the interests of medical science, and he requested a surgical friend, whom he had often consulted with reference to the chronic disease, which at last proved fatal, to be present at the examination, and explain the symptoms under which he had laboured for many years.

